# Elimination of onchocerciasis in Ecuador: findings of post-treatment surveillance

**DOI:** 10.1186/s13071-018-2851-3

**Published:** 2018-04-24

**Authors:** Ángel Guevara, Raquel Lovato, Roberto Proaño, Mario A. Rodriguez-Perez, Thomas Unnasch, Philip J. Cooper, Ronald H. Guderian

**Affiliations:** 1 Instituto de Biomedicina, Universidad Central del Ecuador, Quito, Ecuador; 2 Programa Nacional de Eliminación de la Oncocercosis en el Ecuador, MSP, Quito, Ecuador; 3 Desarrollo Comunitario Vozandes, Hospital Vozandes, Quito, Ecuador; 4 Centro de Biotecnología Genómica, Instituto Politécnico Nacional, Reynosa, Mexico; 5 GHIDR Program, Department of Global Health, College of Public Health, University of South Florida, Tampa, FL 33620 USA; 6 Facultad de Ciencias Médicas de la Salud y la Vida, Universidad Internacional el Ecuador, Quito, Ecuador; 7 Institute of Infection & Immunity, St George’s University of London, London, UK

**Keywords:** Ecuador, Onchocerciasis, Elimination, Transmission

## Abstract

**Background:**

The Esmeraldas focus of onchocerciasis in Ecuador expanded geographically during the 1980s and was associated with severe ocular and skin disease. Mass drug administration (MDA) with ivermectin started in 1991, initially once but later twice a year, in the principle endemic focus followed by all satellite foci. Treatment was stopped in 2009 when entomological assessments determined that transmission of *Onchocerca volvulus* had been interrupted.

**Methods:**

Three years after the cessation of ivermectin treatment in 2012, as defined by the WHO guidelines for onchocerciasis elimination, blackfly collections were done in four sentinel sites in former hyperendemic areas. The presence of infective larvae in local vectors, *Simulium exiguum* and *Simulum quadrivittatum*, was assessed by detection of *O. volvulus* DNA by PCR. Additional flies captured in four extra-sentinel sites located in former hyper- and mesoendemic dispersed isolated areas were also assessed.

**Results:**

The results from 68,310 captured blackflies, 40,114 from four sentinel villages in the previously hyperendemic areas (Corriente Grande, El Tigre, San Miguel on Río Cayapas and Naranjal on Río Canandé) and 28,197 from extra-sentinel locations, were all negative for the presence of *O. volvulus*. These extra-sentinel sites (Hualpí on Río Hoja Blanca, Capulí on Río Onzole, La Ceiba on Río Tululví and Medianía on Río Verde) were included to provide additional evidence of the impact of MDA on the transmission of *O. volvulus* in isolated endemic areas.

**Conclusions:**

Our data indicate that transmission of *O. volvulus* has been stopped in all endemic areas in Ecuador, including all satellite foci outside the main focus. These findings indicate that a strategy of ivermectin distribution twice a year to over 85% of the treatment-eligible population was effective in eliminating the infection from Ecuador in a focus with a highly competent primary vector, *S. exiguum*, and where the infection rates were equal to or greater than observed in many onchocerciasis foci in Africa.

## Background

Onchocerciasis (“river blindness”), caused by the filarial parasite *O. volvulus*, has historically been a major cause of blindness and hindered economic development worldwide [1, 2]. Treatment was revolutionized by the discovery that ivermectin (Mectizan®) was safe and effective for mass distribution, and by the decision by Merck & Co. to donate the drug for the treatment and control of onchocerciasis [1, 2]. Observations on the effects of ivermectin on the transmission of *O. volvulus* in Guatemala [3, 4] raised the possibility that mass distribution of ivermectin at a community-wide level, either annually or semi-annually, might prove sufficient to eliminate the disease through breaking transmission of the parasite. The first programme to implement an elimination rather than a control strategy for onchocerciasis based on ivermectin was the Onchocerciasis Elimination Programme of the Americas (OEPA). OEPA’s strategy was to provide semi-annual treatments with ivermectin at a coverage rate ≥ 85% of all eligible individuals residing in the 13 foci of onchocerciasis in the six endemic countries in Latin America.

By ensuring high coverage rates in the eligible population over a period of several years, it was believed that transmission of the parasite could be suppressed for a long enough period such that the parasite population would eventually be pushed below the transmission breakpoint, and the parasite population would become locally extinct. The World Health Organization (WHO) developed a series of metrics, relying upon entomological and epidemiological indicators to determine when transmission had been suppressed, originally published in 2001 [5] and revised in 2016 [6]. These guidelines envisioned that mass drug administration could be stopped once transmission was determined to have been interrupted, followed by a three to five year period of post-treatment surveillance (PTS) at the end of which entomological surveys would be done to detect recrudescence of transmission. If no evidence for ongoing transmission was observed at this time, it could be concluded that transmission had been eliminated.

**Fig. 1:**
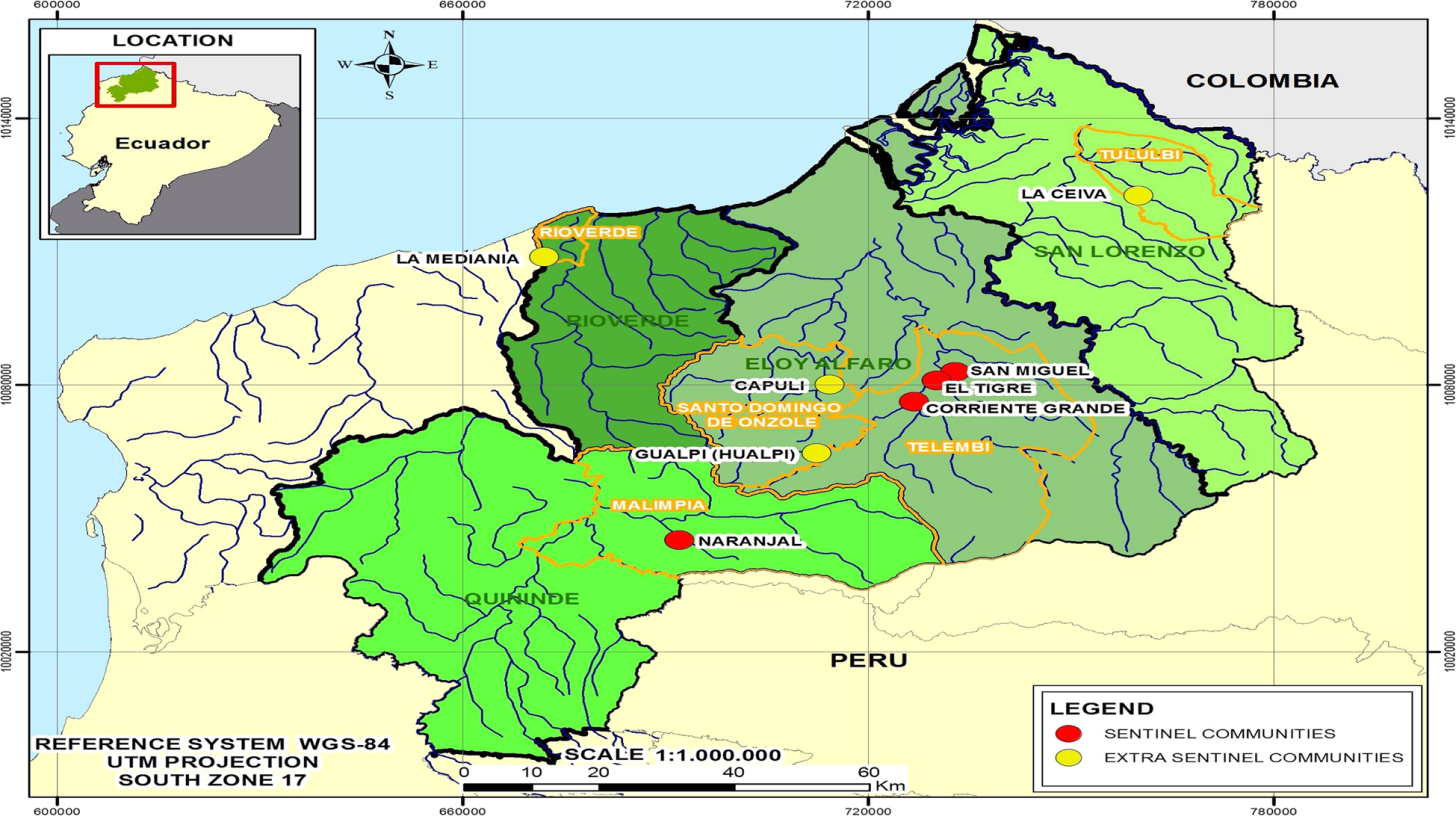
Location of sentinel and extra-sentinel communities include in the post-treatment survey, 3 years after the cessation of MDA

Ecuador is one of six endemic countries for onchocerciasis in Latin America where the infection was confined to a geographically delimited area within the Province of Esmeraldas, located in the northwest region of the country, a rainforest area characterized by complex river systems (Fig. 1). An updated population census in 2008 showed 25,506 people to be living in the endemic area in 119 communities. The vectors in the focus include *S. exiguum* and *S. quadrivittatum*, with the former being the primary vector. *Simulium exiguum* has been shown to be a highly efficient vector for *O. volvulus* [7–11] while *S. quadrivittatum* is much less efficient due to the presence of a cibarial armature that damages the parasites. Epidemiological studies during the 1980s documented a dramatic increase in the intensity of transmission [12–18]. In addition, surveys during the 1980s and 1990s documented the geographical extension of onchocerciasis from the principal focus in the Santiago River Basin to satellite foci other river systems within and outside Esmeraldas Province caused by migrations of *O. volvulus*-infected individuals.

Ivermectin mass drug administration, given either annually or semi-annually, started in 1991 in affected communities and entomological and epidemiological surveys in 2008 indicated that transmission throughout the former endemic area had been interrupted [19]. The National Elimination Programme for Onchocerciasis stopped MDA in the sub-focus on the Rio Santiago in 2008 and in all other foci in 2009, moving into a three-year post-treatment surveillance (PTS) phase to confirm elimination of the infection [5, 19]

This present study presents the results of the PTS entomological surveys done in 2012 at the end of a 3-year PTS period.

## Methods

### Study area

The principal focus was in Esmeraldas Province in the Santiago River Basin, formed by three major river systems of Río Cayapas, Río Santiago and Río Onzole (Fig. 1) [12, 13]. Satellite endemic foci were located on separate river systems [17]: five were found on the rivers, Río Canandé, Río Verde, Río Viche, Río Sucio and Río Tululví; and a small focus was found in the neighboring Province of Santo Domingo de los Tsáchilas. The area has dense tropical vegetation with a high annual rainfall (up to 8000 mm annually). *Simulium exiguum* and *S. quadrivittatum* vectors breed on rocks and vegetation in fast-flowing rivers with a peak biting period between April and June.

The population was comprised largely of two ethnic groups, Chachi Amerindians and Afro-Ecuadorians, living in small riverine settlements. The National Elimination Programme for Onchocerciasis maintained a regularly updated census on the total population using local community health workers who were responsible also for ivermectin treatments. Ivermectin MDA was distributed annually to semi-annually following standard guidelines [2] between 1990 and 2009 with the last dose being given in December 2009. Between 23 and 30 rounds of annual or semi-annual ivermectin MDA was given with high rates of coverage in affected communities [19]. Entomological and serological evaluations, done in 2008 at 4 sentinel sites, showed neither evidence of infection in 48,918 blackflies nor evidence of the presence of *O. volvulus*-specific antibodies in a total of 609 children aged up to 15 years, indicating the interruption of transmission, and leading to the suspension of treatment in 2009 [19]. No ocular microfilariae had been observed in any individual in surveys done after 2000 [19]. All four sentinel sites evaluated in 2009 were evaluated in the present study. Sentinel communities on the Rio Santiago were also hyperendemic prior to ivermectin: MDA was stopped in these communities in 2007, based on an absence of infected flies and seropositive children, and negative parasitological and clinical surveys in 2000 and 2004.

### Sample collection

The four sentinel sites chosen for the final PTS evaluation were formerly hyperendemic villages: Corriente Grande, El Tigre and San Miguel on Río Cayapas, and Naranjal on Río Canandé. These sites had been used for previous surveys [13–16]. Four extra-sentinel sites chosen for PTS evaluation were former hyper- and mesoendemic villages: Hualpí on Río Hoja Blanca, Capulí on Rio Onzole, Medianía on Río Verde and La Ceiba on Río Tululví (Fig. 1). The four extra-sentinel communities were included in the PTS evaluation in support of the data collected in hyperendemic sentinel communities and to ensure that the findings in the peripheral geographical endemic areas were representative of the transmission status of the whole endemic zone.

**Fig. 2:**
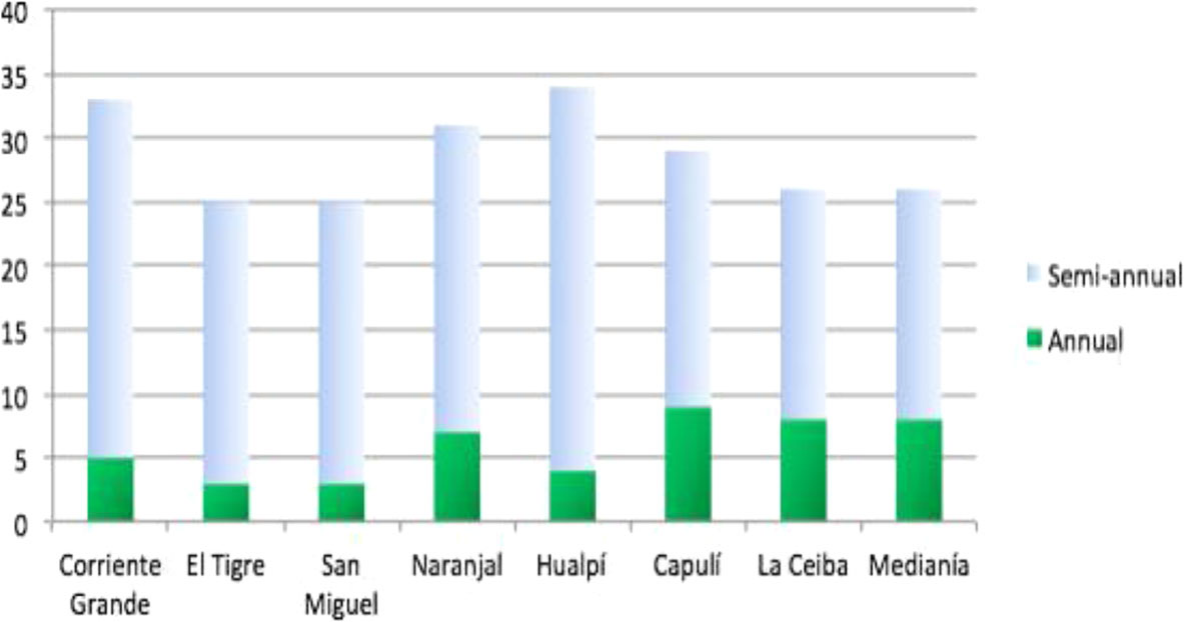
Ivermectin treatments received by the communities involved in the study: the number of annual-only treatments (solid green bars) and the number of semi-annual treatments administered (blue bars). Corriente Grande, El Tigre, San Miguel and Naranjal are sentinel village sites in the main focus; Hualpí, Capulí, La Ceiba and Medianía are extra-sentinel villages

The eight communities selected for PTS, received between 25 and 35 annual or semi-annual treatments with ivermectin from 1990 to 2009 when MDA was stopped [19] (Fig. 2). Flies were collected using standard methods [15] from 08:00–17:00 h each day for 8 days per month in April-June 2012. Collection sites in sentinel communities were those where human-fly contact was greatest and were generally shady areas close to riverbanks within the communities. Flies were collected for 50 min each hour, allowing a 10 min break, then sorted according to species (*S. exiguum* and *S. quadrivittatum*), divided into pools containing a maximum of 50 individuals of each species per pool, placed in isopropanol and stored at room temperature until analyzed by PCR.

### Entomological (PCR) testing

Heads and bodies from the collected flies were separated by freezing, agitation and separation through a 25 mesh sieve [20]. DNA was extracted following proteinase K digestion, organic extraction and adsorption to a silica matrix [21]. Pools were processed in groups of 12, with each group consisting of 11 fly pools and one sham extraction (i.e. containing PCR reagents in the absence of flies) serving as an internal negative control. The resulting DNA preparations were used as templates in a PCR assay targeting an *O. volvulus*-specific repeated sequence (O-150 PCR). The PCR products were detected by PCR-ELISA [20, 21]. Pools were regarded as putatively positive for *O. volvulus* DNA when ELISA values were equal to or greater than the mean plus three standard deviations of the values obtained from 10 negative control wells run on each plate. Putatively positive DNA samples were then re-tested in an independent PCR reaction. Samples that were positive in both assays were classified as “confirmed positives”. Pools of bodies were initially screened, as bodies contain early stage larvae (microfilarial and L2 stages) are the most sensitive indicator of parasite-vector contact. The prevalence of flies containing immature stages is 2-fold higher than the prevalence of flies containing infective third-stage larvae (L3) in *S. exiguum* and 20-fold higher in *S. quadrivittatum* [22]. DNA from head pools were screened if evidence for parasite-vector contact was observed in the screens of the body pools to obtain an estimate of the prevalence of flies containing L3.

### Statistical methods

The upper limit of the 95% confidence interval for infection rate of flies carrying *O. volvulus* parasites was calculated using a Bayesian algorithm from Poolscreen v.2.0. Upper limits for the 95% confidence interval for the seasonal transmission potential were calculated as the products of the upper limits of the 95% confidence intervals for the seasonal biting rate, the prevalence of infected flies and the mean number of infective stage larvae per fly, as previously described [22]. In undertaking these calculations, the mean number of L3s per infective fly was taken as 1, as reported to be the case in areas where there are effective control measures.

## Results

### Blackfly populations

A total of 68,311 flies were collected from the eight communities in April and June (i.e. during the peak transmission season in the Esmeraldas focus) in 2012. *Simulium. exiguum* made up approximately 75% of flies and was the dominant species collected in all communities except for La Ceiba (Table 1).

**Table 1:** Post-treatment surveillance blackfly collections at sentinel and extra-sentinel communities in 2012: total number of flies examined

Community	Type	*S. exiguum*	*S. quadrivittatum*
Corriente Grande	Sentinel	8272	1604
El Tigre	Sentinel	4188	3565
San Miguel	Sentinel	7024	426
Naranjal	Sentinel	14,639	396
Capulí	Extra-sentinel	3182	5
Hualpí	Extra-sentinel	6972	3672
Medianía	Extra-sentinel	7212	1
La Ceiba	Extra-sentinel	3	7150
Total		51,492	16,819

### PCR detection of infected flies

Flies were grouped by community and species into pools and screened for the presence of *O. volvulus* DNA by PCR. None of the pools were found to be positive giving a point estimate for the prevalence of infection in the fly population (and the associated point estimate of the seasonal transmission potential) of zero (Table 2). The upper limit of the 95% confidence interval for the rate of *O. volvulus* infected flies ranged from 0.1 to 1.2 per 2000 flies sampled in the different communities (depending upon the number of flies collected from each community). However, the upper limit of the infection rate in the fly population in the focus as a whole was 0.1 per 2000 flies sampled (Table 2). Similarly, the upper limit of the 95% confidence interval for the seasonal transmission potential for each community ranged from 6.4 to 17.4, and was 1.0 for the focus as a whole (Table 2). When the data were analyzed by species, the upper limit of the 95% confidence interval for the infection rates in *S. exiguum* and *S. quadrivittatum* was 0.1/2000 and 0.2/2000 flies, respectively (Table 3). The upper limit of the 95% confidence interval for the seasonal transmission potential for both species was less than one L3 per year (Table 2).

**Table 2:** Prevalence of infected flies and seasonal transmission potential in sentinel and extra-sentinel communities, 2012

Community	Flies screened	Pools screened	Positive pools	Rate of infected flies^a^ (CI)^b^	Seasonal biting rate (CI)^b^	Seasonal transmission potential (CI)^b^
Corriente Grande	9876	236	0	0 (0–0.39)	56,867 (53,162–60,825)	0 (0–11.1)
El Tigre	7753	216	0	0 (0–0.49)	40,797 (38,821–42,872)	0 (0–10.0)
San Miguel	7450	200	0	0 (0–0.51)	27,833 (25,218–30,708)	0 (0–7.1)
Naranjal	15,035	328	0	0 (0–0.26)	133,890 (117,616–152,396)	0 (0–17.4)
Capulí^c^	3187	81	0	0 (0–1.20)	10,739 (9929–11,609)	0 (0–6.4)
Hualpí^c^	10,644	226	0	0 (0–0.40)	65,127 (60,981–69,550)	0 (0–11.7)
Medianía^c^	7213	154	0	0 (0–0.50)	24,615 (21,388–28,305)	0 (0–6.5)
La Ceiba^c^	7153	151	0	0 (0–0.50)	38,348 (36369–40,432)	0 (0–10.2)
Total	68,311	1592	0	0 (0–0.10)	34,117 (32,796–35,490)	0 (0–1.0)

^a^Number infected flies per 2000 flies sampled

^b^Shown are point estimates and 95% confidence intervals (CI)

^c^Extra-sentinel sites

**Table 3:** Rate of infected flies and seasonal transmission potential in *Simulium exiguum* and *Simulium quadrivittatum* in 2012

Species	Flies caught	Flies screened	Positive pools	Rate of infected flies^a^ (CI)^b^	Seasonal biting rate (CI)^b^	Seasonal transmission potential (CI)^b^
*S. exiguum*	51,492	1225	0	0 (0–0.1)	16,275 (14,931–17,731)	0 (0–0.6)
*S. quadrivittatum*	16,819	367	0	0 (0–0.2)	4655 (4217–5128)	0 (0–0.5)

^a^Number of infected flies per 2000 flies sampled

^b^Shown are point estimates and 95% confidence intervals (CI)

## Discussion

Following the description of the first case of onchocerciasis in Ecuador in 1980 [23], the principal endemic focus in Province of Esmeraldas has been extensively described. Up until 1991, disease control relied on a systematic programme of surgical nodulectomies that had limited success in reducing infection indices in the hyperendemic foci. A mass drug administration programme with annual or semi-annual treatments with ivermectin was initiated in 1991 in which coverage of over 85% of the eligible population was consistently achieved for much of the 16-year period of the programme. Findings of entomological and serological surveys indicated that transmission had been interrupted and ivermectin MDA was stopped in 2009 [19]. The present study provides the findings of post-treatment surveillance (PTS) and shows, after at least three years of stopping treatment, no active transmission of *O. volvulus* infection in any of the sampled blackfly populations.

Post-treatment surveillance (PTS) was done in 2012 in accordance with the 2001 WHO guidelines for the verification of onchocerciasis elimination [5]. This assessment included fly collections in sentinel sites in the formerly hyperendemic region in the Santiago River Basin: none of the 40,114 flies collected were found to be positive for *O. volvulus* by PCR. Extra-sentinel sites were surveyed to ensure that transmission had been successfully contained: none of the 28,197 flies collected from these sites were positive for *O. volvulus* DNA. Extra-sentinel sites were included in PTS because previous studies had shown that infected individuals who had migrated from the Santiago River Basin focus to these communities had led to the establishment of new satellite foci in areas where a suitable vector was present.

The Ecuadorian National Elimination Programme for Onchocerciasis has concluded that after over 15 years of annual and semi-annual mass drug administration with ivermectin in the endemic foci in Ecuador, *O. volvulus* transmission had been eliminated in Ecuador in accordance with past [5] and current [6] WHO criteria for the elimination of this infection. Based on the well-documented history of the MDA programme in Ecuador and the results of entomological and clinical surveys that showed no evidence of transmission up to the halting of treatment and during the three years of PTS, the Ecuador programme requested external verification of the interruption of transmission and approval of the status of elimination of *O. volvulus* transmission. The WHO certified the elimination of onchocerciasis from Ecuador in September 2014. This success was achieved in an endemic focus with special challenges for disease control and elimination. First, the dominant vector, *S. exiguum*, was a particularly voracious and effective transmitter of the parasite [8–11, 21, 22], and secondly, the onchocercal skin and eye disease in this focus was arguably the most severe form in the Americas [13, 14, 18, 24].

Among the important factors contributing to this achievement were: (i) the effective leadership and operational expertise of the programme combined with constant and close interactions with affected communities; and (ii) a semi-annual treatment schedule combined with high coverage rates. Key actors in the success of this treatment-based strategy were community health workers (CHWs), supported closely by the National Elimination Programme for Onchocerciasis team and integrated within multi-disciplinary health teams providing a broad range of health services and educational activities to endemic communities. Such activities ensured high rates of treatment coverage long after onchocerciasis was perceived as a health problem in endemic communities. Treatments were directly observed and censuses were updated semi-annually by CHWs with individualized information on numbers of treatments received. CHWs were supported in their activities directly by local Ministry of Public Health and NGO health personnel. Despite the remoteness of many of the residents of the area due to the extensive river systems, the national team placed considerable emphasis on maintaining up-to-date knowledge of the changing situation with regards to migration, coverage rates and the provision of medical care. These factors undoubtedly contributed to the success of the programme.

An important consideration for any disease elimination programme is the possibility of spread of the infection to new areas through migrations of uninfected individuals into areas where transmission is still active or through migrations of infected individuals to unaffected areas where suitable vectors are present. In addition, previous residents of an endemic area who have not received treatment but who carry the infection, could return to an area where transmission has been suppressed and reintroduce infection, thus re-establishing transmission. Key to the establishment of new focus is the presence of a competent vector(s) capable of initiating and maintaining transmission. The vectorial capacities of *Simulium* sp. have been studied in several infection foci in Ecuador including studies of cytotaxonomy [25–29]. Satellite foci were identified through a combination of methods that included anthropological evaluations of migration patterns within the Santiago River Basin focus and community censuses to identify origins of new in-migrants and destinations of out-migrants. Based on these findings, the programme identified potential regions that could be potential sites of new foci and did surveys in all these sites between 1985 and 1995 that included entomological surveys for the presence of potential vector species of *Simulium* blackflies and community surveys for the presence of infection. These studies identified satellite foci within and adjacent to Esmeraldas Province to which infected Chachilla Amerindians had migrated and demonstrated autochthonous transmission in some [17, 30, 31]. Through these studies it was established that as few as 4–6 *O. volvulus*-infected migrants could set up a new focus of infection, an event most likely determined by factors such as number of infected migrants, their respective microfilarial loads, and the competence of the local vector for parasite transmission [30].

These studies showed, however, that the areas outside the established foci capable of supporting parasite transmission were limited. Those identified as new foci were monitored closely by the national programme both before and after MDA [16, 17]. Mathematical models of onchocerciasis transmission have suggested that the force of infection is strongly influenced both by the competence of the local vector and the amount of human-vector contact, measured by the biting rate [21, 32]. The higher the biting rate and the more competent the vector, the greater the force of infection and the more difficult to eradicate the infection from a focus. In the Esmeraldas focus, the primary vector *S. exiguum*, is an efficient vector, equaling the competence of forest *S. damnosum* (*s.l.*) cytotypes in Africa, in terms of the percentage of flies developing infective-stage larvae and the numbers of larvae per infected fly [9]. In addition, *S. exiguum* has been estimated to have high biting rates estimated at 385 and 733 third-stage larvae/person in the communities of San Miguel and El Tigre, respectively [33]. Together, these factors suggested that onchocerciasis would, at least theoretically, prove to be difficult to eliminate from Ecuador.

Our data provide strong evidence that transmission has not resumed in Ecuador in the three years between the last ivermectin treatment and the time of this PTS entomological survey. Current 2016 WHO guidelines for the certification of the elimination of transmission of *O. volvulus* [6] state that there should be < 1 infective fly per 2000 (0.05%) flies tested assuming that 50% of flies are parous. To reach this operational threshold, a minimum sample size required to have enough power to detect a statistically significant prevalence of infective flies lower than 0.05% (i.e. not included in the 95% Cl), given that no infective fly may be found, is at least 6000 flies per community [6]. In this survey, no evidence of infection was seen in an analysis of over 60,000 flies collected from eight communities in the Esmeraldas focus. This included over 50,000 *S. exiguum*, the primary vector for *O. volvulus* in the focus, and over 10,000 *S. quadrivittatum*, the secondary vector. Taken together, the upper bound of the 95% confidence interval for the prevalence of flies carrying *O. volvulus* was ten-fold less that the 1/2000 cut-off established by OEPA. These data strongly support the assertion that transmission of *O. volvulus* has been eliminated from Ecuador.

Before stopping ivermectin MDA, the Programme established a surveillance system in which onchocerciasis became a mandatory notifiable disease within the national surveillance system of the Ecuadorian Ministry of Public Health. At the start of PTS, which began in the Rio Santiago focus in 2008 and in the remaining endemic areas in 2009, communities were notified of visits by programme staff through the network of CHWs. Each formerly endemic community was visited by programme staff performing three basic activities: providing (i) information and education about why ivermectin treatment was being stopped through community assemblies; (ii) clinical palpation for potential onchocercal nodules; and (iii) medical and dental care provided by a multidisciplinary team from the Ministry of Health. These visits were done annually over the three-year PTS period. CHWs attended workshops on PTS and were responsible for updating community censuses and detection for suspicious nodules or clinical disease indicating recrudescence of onchocerciasis. Suspicious clinical findings were discussed at monthly meetings of CHWs at a central location with programme staff. Similarly, annual treatments with albendazole were provided through CHWs in endemic communities for the treatment of intestinal parasites. Suspect nodules (all confirmed not to be of onchocercal origin) were removed at annual surgical field clinics. Furthermore, special bilingual educational materials and a newsletter (‘Hora Onco’) were distributed to each household in the communities to notify the communities about planned visits, community assemblies, and educational workshops. The combination of these continuous activities ensured that the communities were well informed about the cessation of ivermectin MDA and the need for continuing surveillance for recrudescence of infection.

## Conclusions

The finding that the interruption in vector transmission has been maintained over a period of three years since the cessation of treatment in 2009 strongly supports the conclusion that Ecuador has indeed eliminated onchocerciasis. Thus, these results strongly suggest that the elimination of microfilarial infection by ivermectin MDA, perhaps optimally when administered every six months, coupled with any residual effects these recurrent treatments might have on adult worm survival, can be achieved even under circumstances of unusually high annual biting rates by a competent vector.

## Abbreviations

CHWs: Community health workers
DNA: Deoxyribonucleic acid
ELISA: Enzyme-linked immunosorbent assay
L3: Infective larvae
MDA: Mass drug administration
MSP: Ministerio de Salud Pública del Ecuador
NGO: Non-governmental organization
OEPA: Onchocerciasis Elimination Programme of the Americas
PAHO: Pan American Health Organization
PCC: Programme coordinating committee
PCR: Polymerase chain reaction
PTS: Post-treatment surveillance
WHO: World Health Organization

## References

[1] Cupp EW, Mackenzie CD, Unnasch T (2011). The importance of ivermectin to human onchocerciasis: past, present, and the future. Res Rep Trop Med.

[2] Thylefors B (2008). The Mectizan Donation Program (MDP). Ann Trop Med Parasitol.

[3] Cupp EW, Bernardo MJ, Kiszewski AE, Collins RC, Taylor HR, Aziz MA, Greene BM (1986). The effects of ivermectin on transmission of *Onchocerca volvulus*. Science.

[4] Collins RC, Gonzales-Peralta C, Castro J, Zea-Flores G, Cupp MS, Richards FO, Cupp EW (1992). Ivermectin: reduction in prevalence and infection intensity of *Onchocerca volvulus* following biannual treatments in five Guatemalan communities. Am J Trop Med Hyg.

[5] World Health Organization. Certification of elimination of human onchocerciasis: criteria and procedures. Geneva: World Health Organization; 2001. http://apps.who.int/iris/bitstream/handle/10665/66889/WHO_CDS_CPE_CEE_2001.18b.pdf?%20sequence=1&isAllowed=y%20%20p.%201%E2%80%9329 p. 1–29. Accessed 4 Apr 2018.

[6] WHO (2016). Guidelines for stopping mass drug administration and verifying elimination of human onchocerciasis. Criteria and procedures.

[7] Shelley AJ, Arzube M (1985). Studies on the biology of Simuliidae (Diptera) at the Santiago onchocerciasis focus in Ecuador, with special reference to the vectors and disease transmission. Trans R Soc Trop Med Hyg.

[8] Wetten S, Collins RC, Vieira JC, Marshall C, Shelley AJ, Basanez MG (2007). Vector competence for *Onchocerca volvulus* in the *Simulium* (*Notolepria*) *exiguum* complex: cytoforms or density-dependence. Acta Trop.

[9] Collins RC, Lehmann T, Vieira JC, Guderian RH (1995). Vector competence of *Simulium exiguum* for *Onchocerca volvulus*: implications for epidemiology of onchocerciasis. Am J Trop Med Hyg.

[10] Shelley AJ, Procunier WS, Arzube M (1986). Direct incrimination of *Simulium exiguum* cayapa form as a vector of *Onchocerca volvulus* in Ecuador. Trans R Soc Trop Med Hyg.

[11] Arzube M, Shelley AJ (1990). Seasonal variation in onchocerciasis transmission in the Santiago focus of Ecuador. Trop Med Parasitol.

[12] Guderian RH, Leon LA, Leon R, Corral F, Vazconez C, Johnston TS (1982). Report on a focus of onchocerciasis of Esmeraldas Province of Ecuador. Am J Trop Med Hyg.

[13] Guderian RH, Swanson D, Carrillo RD, Proaño S, Molea J, Swanson WL (1983). Onchocerciasis in Ecuador. I. Prevalence and distribution in the Province of Esmeraldas. Tropenmed Parasit.

[14] Guderian RH, Molea J, Swanson D, Proaño SR, Carrillo RD, Swanson WL (1983). Onchocerciasis in Ecuador. II. Epidemiology of the endemic foci in the Province of Esmeraldas. Tropenmed Parasit.

[15] Guderian RH, Beck BJ, Stone DJ, Isabel K, Mackenzie CD (1988). Onchocerciasis in Ecuador: recent observations in the Province of Esmeraldas. J Trop Med Hyg.

[16] Guderian RH, Beck BJ, Proaño SR, Mackenzie CD (1989). Onchocerciasis in Ecuador. 1980–1986: epidemiological evaluation of the disease in the Esmeraldas Province. Eur J Epidemiol.

[17] Guderian RH, Shelley AJ (1992). Onchocerciasis in Ecuador: the situation in 1989. Mem Inst Oswaldo Cruz.

[18] Cooper PJ, Proaño SR, Beltran C, Anselmi M, Guderian RH (1995). Onchocerciasis in Ecuador: ocular findings in *Onchocerca volvulus* infected individuals. Br J Ophthalmol.

[19] Lovato R, Guevara A, Guderian R, Proaño R, Unnasch T, Criollo H (2014). Interruption of infection transmission in the onchocerciasis focus of Ecuador leading to the cessation of ivermectin distribution. PLoS Negl Trop Dis.

[20] Rodríguez-Pérez MA, Lilley BG, Domínguez-Vázquez A, Segura-Arenas R, Lizarazo-Ortega C, Mendoza-Herrera A (2004). Polymerase chain reaction monitoring of transmission of *Onchocerca volvulus* in two endemic states in Mexico. Am J Trop Med Hyg.

[21] Guevara AG, Vieira JC, Lilley BG, Lopez A, Vieira N, Rumbea J (2003). Entomological evaluation by pool screen polymerase chain reaction of *Onchocerca volvulus* transmission in Ecuador following mass Mectizan distribution. Am J Trop Med Hyg.

[22] Katholi CR, Toe L, Merriweather A, Unnasch TR (1985). Determining the prevalence of *Onchocerca volvulus* infection in vector populations by polymerase chain reaction screening of pools of black flies. J Infect Dis.

[23] Carvajal HL, Zerega F (1980). La oncocercosis en Ecuador. Primer caso demostrado. Rev Ecu Hig Med Trop.

[24] Hay RJ, Mackenzie CD, Guderian R, Noble WC, Proano JR, Williams JF (1989). Onchodermatitis - correlation between skin disease and parasitic load in an endemic focus in Ecuador. Br J Dermatol.

[25] Procunier WS, Shelley AJ, Azube M (1987). Cytological identification of *Simulium oyapockense manabi* form (Diptera: Simuliidae): a potential vector of onchocerciasis in Ecuador. Trop Med Parasitol.

[26] Procunier WS (1989). Cytological approaches to simuliid biosystematics in relation to the epidemiology and control of human onchocerciasis. Genome.

[27] Shelley AJ, Charalambous M, Azube M (1990). *Onchocerca volvulus* development in four *Simulium exiguum* cytospecies in Ecuador. Bull Soc Fr Parasitol.

[28] Charalambous M, Ready PD, Shelley AJ, Arzube M, Lowry GA (1993). Cytological and isoenzyme analysis of the Bucay and Quevedo cytotypes of the onchocerciasis vector *Simulium exiguum* (Diptera: Simuliidae) in Ecuador. Mem Inst Oswaldo Cruz.

[29] Charalambous M, Arzube M, Lowell S (1998). Cytogenetic analysis of a new sub-complex of *Simulium exiguum* (Diptera: Simuliidae) in Amazonian Ecuador. Bull Entomol Res.

[30] Guderian RH, Proaño S, Milhon JL, Johnson DE, Herdoiza VM (1987). Oncocercosis en el Ecuador: epidemiologia de los seis focos satelites de la provincia de Esmeraldas. Rev Méd Vozandes.

[31] Cooper PJ, Mancero T, Espinel M, Sandoval C, Lovato R, Guderian RH, Nutman TB (2001). Early human infection with *Onchocerca volvulus* is associated with an enhanced parasite-specific cellular immune response. J Infect Dis.

[32] Basanez MG, Richardez-Esquinca J (2001). Models for the population biology and control of human onchocerciasis. Trends Parasitol.

[33] Vieira JC, Brackenboro L, Porter CH, Basáñez MG, Collins RC (2005). Spatial and temporal variation in biting rates and parasite transmission potentials of onchocerciasis vectors in Ecuador. Trans R Soc Trop Med Hyg.

